# From Program to Policy: Expanding the Role of Community Coalitions

**Published:** 2007-09-15

**Authors:** Anne Hill, Jill Guernsey De Zapien, Lisa K Staten, Deborah Jean McClelland, Martha Moore-Monroy, Joel S Meister, Rebecca Garza, JoJean Elenes, Victoria Steinfelt, Ila Tittelbaugh, Evelyn Whitmer

**Affiliations:** Mel and Enid Zuckerman College of Public Health, University of Arizona; Mel and Enid Zuckerman College of Public Health, University of Arizona, Tuscon, Arizona; Mel and Enid Zuckerman College of Public Health, University of Arizona, Tuscon, Arizona; Mel and Enid Zuckerman College of Public Health, University of Arizona, Tuscon, Arizona; Mel and Enid Zuckerman College of Public Health, University of Arizona, Tuscon, Arizona; Mel and Enid Zuckerman College of Public Health, University of Arizona, Tuscon, Arizona; Migrant Health Promotion, REACH 2010 Promotora Community Coalition, Progreso, Texas; Mariposa Community Health Center, Nogales, Arizona; University of Arizona Cooperative Extension, Yuma County, Yuma, Arizona; Nogales Special Action Group, Nogales, Arizona; University of Arizona Cooperative Extension, Family and Consumer Sciences, Community Health Programs — Cochise County, Sierra Vista, Arizona

## Abstract

**Background:**

Diabetes mortality at the United States–Mexico border is twice the national average. Type 2 diabetes mellitus is increasingly diagnosed among children and adolescents. Fragmented services and scarce resources further restrict access to health care. Increased awareness of the incidence of disease and poor health outcomes became a catalyst for creating community-based coalitions and partnerships with the University of Arizona that focused on diabetes.

**Context:**

Five partnerships between the communities and the University of Arizona were formed to address these health issues. They began with health promotion as their goal and were challenged to add policy and environmental change to their objectives. Understanding the meaning of *policy* in the community context is the first step in the transition from program to policy. Policy participation brings different groups together, strengthening ties and building trust among community members and community organizations.

**Methods:**

Data on progress and outcomes were collected from multiple sources. We used the Centers for Disease Control and Prevention's Racial and Ethnic Approaches to Community Health (REACH) 2010 Community Change Model as the capacity-building and analytic framework for supporting and documenting the transition of coalitions from program to policy.

**Consequences:**

Over 5 years, the coalitions made the transition, in varying degrees, from a programmatic focus to a policy planning and advocacy focus. The coalitions raised community awareness, built community capacity, encouraged a process of "change in change agents," and advocated for community environmental and policy shifts to improve health behaviors.

**Interpretation:**

The five coalitions made environmental and policy impacts by engaging in policy advocacy. These outcomes indicate the successful, if not consistently sustained, transition from program to policy. Whether and how these "changes in change agents" are transferable to the larger community over the long term remains to be seen.

## Background

The impact of chronic disease on Southwest border communities is stark. The United States–Mexico border is medically underserved and considered one of the poorest regions in the country (1). Along the border, the rate of diabetes mortality is two times greater than the national average (2). In addition, type 2 diabetes mellitus is increasingly being diagnosed among children and adolescents (3). Health risk behaviors, such as lack of physical activity and poor nutrition, are linked to increased risk for many chronic diseases. Fragmented services and scarce resources further restrict the access to health care for residents.

These largely Hispanic border communities experienced pronounced health changes during the 1990s and 2000s (4,5), including an increase in obesity among adults and children and an increase in diabetes rates and diabetes-related complications. These changes were compounded by a lack of resources to address these issues. Increased awareness of the incidence of disease and poor health outcomes became a catalyst for creating community-based coalitions and university–community partnerships that focus on diabetes.

Reversing border communities' poor health trends requires effective long-term primary prevention programs and comprehensive, community-oriented approaches that target the local environment — social, political, and cultural. Policy action is a means by which community coalitions can create sustainable environmental, policy, and behavior changes.

The impetus for systems change in chronic disease prevention came from extensive discussions among members of the University of Arizona (UA) Prevention Research Center's Community Action Board (CAB). The CAB consisted of representatives from the four Arizona–Mexico border communities and two tribal nations as well as nongovernmental organizations and health departments, both state and local. After completing a month-long strategic planning process, in 2000 the CAB concluded that, although awareness of chronic disease and its complications had increased in various border communities, efforts should be increased to develop community capacity to make a greater long-term impact on chronic disease prevention and control through policy and environmental change. The burden of diabetes at the border remained the central theme, but the CAB wanted to emphasize developing models that went beyond health programs to focus on systems change. CAB members believed that systems change would lead to sustainable health behavior improvements among individuals and communities.

This article describes the process by which five community coalitions moved from a programmatic to a policy focus and demonstrates how these coalitions used community-based participatory approaches to promote systems change at the local level. This change in approach is both cause and effect of framing health and illness as a communitywide concern.

## Context

The UA partners promoted the CAB's agenda in the five border communities. These partnerships worked because a long history of collaboration, in some cases for over 20 years, existed between the UA and these communities. On the basis of these strong ties, the coalitions asked the UA to be active partners in helping to set coalition agendas, asking the UA to share its expertise in policy development. UA partners became more than just outside technical experts; indeed, they were equal members of the group and full participants. The UA collaborated with coalitions to build local capacity in health promotion and policy advocacy to prevent chronic diseases and complications (6). Through these partnerships, coalition members gained skills in identifying funding sources and grant writing as well as understanding how policies are developed and implemented.

Incorporating policy change into coalitions' initial health program orientation is a challenge. Coalition members find that the transition from program to policy is not simple. Members first work toward understanding policy in a community context of resource allocation and local power centers, and then they move toward developing and implementing an action plan. In addition, members learn advocacy skills as well as how to expand coalition membership to include new stakeholders. This process can be cyclical as new members join the coalition or new policy issues arise. Time needed to educate coalition members and address policy issues can be considerable and is often seen as a barrier to program implementation. Thus, the transition process can be frustrating, especially when broader policy issues are not part of the traditional health promotion culture (7).

Understanding the meaning or definition of *policy* in the community context is the first step in the transition phase. In our experience, *policy* in the community tends to be broadly defined as an institutionalized, organizationally legitimized, ongoing set of regulations, directives, or resource allocations that provide sustained support for programs. Thus, budgetary changes in the local government or schools that support new programs, regulations enacted by local public agencies, and formal changes in a local agency's operations qualify as policy changes.

When coalitions engage in policy advocacy, they are best able to make changes by encouraging broad civic participation and securing community buy-in (6,8). At the local level, policy participation brings different groups together, strengthening ties and building trust among community members and community organizations. The process also requires partners to share energy and resources to address one or more health problems, which may reduce competition for limited funds (7).

## Methods

### REACH 2010 model

We applied the Racial and Ethnic Approaches to Community Health (REACH) 2010 Community Change model, developed by the Centers for Disease Control and Prevention (CDC), as a framework for situating and assessing the shift in coalition focus from program to policy. The REACH 2010 model (Figure) is a logic model that leads through several stages from creating community awareness of an issue such as diabetes to the long-term impact of improving health status. The driving force of change is a series of "targeted actions" such as programs in the community at both the individual and community level that focus on the issue. Among the five border coalitions, some or all of the following programmatic targeted actions were employed: walking clubs; nutrition classes; healthful food demonstrations in grocery stores; health education curriculum development at schools; expanded school physical education programs; use of the CDC's School Health Index (9), including development of school health teams to apply the index; patient self-management classes; and quality improvement initiatives for clinical care of diabetes. This article reports on the policy-oriented targeted actions. These actions target the "changes in change agents" and "environmental shift (i.e., policy)" levels of the REACH 2010 model (6).

**Figure.**

**Figure.**
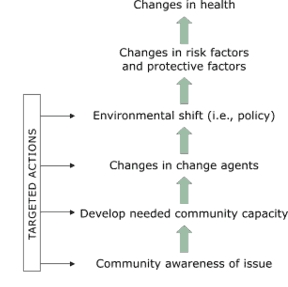
The Racial and Ethnic Approaches to Community Health (REACH) 2010 Model of Change from the Centers for Disease Control and Prevention, adapted by the Southwest Center for Community Health Promotion (6).

The model posits that, among other changes, community change agents will change in order to bring about environmental/policy shifts. In our partner communities, policy involvement gave most coalition members their first chance to work directly with local governments (10). Measurable changes in the knowledge, attitudes, beliefs, and behaviors of coalition members and of local leaders (e.g., school board members, city council members, police officers) regarding health as a community issue would constitute "change in change agents" according to the REACH 2010 model (6). Change agents then become advocates for making environmental shifts and policy changes in the community. These shifts include altering local government budgets for more parks and walking trails or creating nutrition and wellness policies for local public schools. After these stages have been reached, one would expect to observe changes in risk and protective factors and in health behaviors, followed ultimately by changes in health status (11). This is a model for long-term change, a process that might require a decade or more (6,10).

### Data collection

Data from multiple sources were collected for this study. These included, in addition to the initial literature review, coalition meeting minutes, participant observation by UA researchers, and coalition member interviews. We conducted rigorous content analysis of the meeting minutes and interviews to identify how the goals and activities of the five coalitions changed over time. During this analysis, our objective was to look for key variables, including building community awareness through organization of community forums on nutrition and physical activity or through presentations to parents and teachers on community health problems (12). Other key variables included an increase in community capacity, which involved initiating and completing a strategic planning process, implementing an action plan, meeting with local leaders or government officials to discuss the need for safer walkways and more parks, or meeting with schools to improve school nutrition and physical activity regulations. Finally, community partners participated in the writing of this article to ensure that the interpretations of all stakeholders were accurately represented.

### Community awareness and capacity

On the basis of the CAB directive, UA partners promoted the idea of making systemic changes to improve community health behaviors among the community coalitions. UA partners saw their role as facilitating the transition of the community coalitions from a purely program focus to one that gave primacy to policy-related objectives. Community coalition members agreed that changes in their local environment, laws or regulations, and resource allocation were necessary.

The process of changing community change agents (transition from program to policy) took place over a 5-year period for most of the coalitions. First, the coalitions increased their awareness and knowledge of the health problems facing their communities. This learning process led to an increasing awareness of how policy fit into the larger picture of changing health behaviors and improving the community's health.

In some cases the UA partners worked directly with coalition members to help them gain confidence in the process and learn new advocacy skills. The UA partners also assisted in identifying priorities as either programs or policies by adapting an "all on the wall" tool created by the Institute for Cultural Affairs (13). The exercise required that coalition members brainstorm what they would like to see happen in the community. Programmatic versus policy ideas were discussed, and members were encouraged to focus on policy initiatives. All policy suggestions were written on a board. Members were given five stickers to vote on the ideas. Members could use all five to indicate interest in one idea or divide the stickers up among several ideas.

The UA partners also created a "Program/Policy" card game consisting of typical community situations that could be program or policy or some mix. This game can be adapted to any health issue in any community. The five border coalitions all played it in both English and Spanish. A typical game had the coalition members break into small groups. Each group was then given two to four cards. Each card listed one item (e.g., "Start a walking club in your neighborhood," "Get the schools to open their playgrounds for walking and exercise every night") with one side in English and the other in Spanish. Each group was asked to come to agreement about whether their card(s) described a program or a policy. After time for discussion, each group reported on their choice and the reasons for their choice. More discussion followed, as the larger group debated why some items were programmatic, others were policy, and still others were ambiguous depending on the presence or absence of certain conditions, such as sponsorship commitment.

## Consequences

### Change in change agents and environmental/policy shifts

Community coalition members recognized that health, as a policy issue, extended beyond the purview of the county health department. They decided to reach out to various new change agents, such as elected officials, business leaders, law enforcement, members of the faith community, and educational leaders, who would contribute different perspectives on the community. Convincing these change agents to participate in a public health activity was sometimes difficult, because many of them were unsure of the link between their expertise and health in the community.

As the policy focus became clearer, the coalitions identified and prioritized major environmental/policy issues in the communities, developed goals and action plans, and proceeded to recruit new stakeholders, if possible (6,10). Coalition members worked hard at making the link between improvement in community health behaviors and the involvement of professionals in parks and recreation, the local newspaper, and law enforcement (10).

Timing of initiatives and the presence of a policy "window of opportunity" were crucial to the success of the transition process (14). The process of transition from program to policy varied, with each coalition moving at its own pace. In some cases, coalition members recognized that working on a particular policy issue was timely and salient to discussions and interests in the community, although in other coalitions, persistence and patience contributed to a willingness to wait until a window of opportunity was available to act. All coalitions, however, were beginning to recognize the impact of systemic conditions on the health of the community and had to determine whether and to what extent a window of opportunity existed that could increase the chances of success. The results of this process and the resulting actions are presented in the Table. The individual coalitions and their achievements are described below.

### REACH 2010 *Promotora* Community Coalition

During the planning year of a REACH 2010 grant from CDC in 1999, the Michigan-based program, Migrant Health Promotion, Inc, strengthened their office in the Texas Rio Grande Valley to improve the health conditions of residents living in the area. Many residents subsisted in precarious conditions, having little job security or access to health care. To better understand the needs of the community, the organization held town hall meetings in three valley communities. Community members voiced their desire to address diabetes because the disease was affecting 25% of valley residents. Interventions targeted activities in communities, schools, and health clinics.

After receiving the second phase of the grant, during 2000 and 2007 the *Promotora* Community Coalition implemented a community-driven diabetes prevention and control intervention in three rural Texas communities in the lower Rio Grande Valley. The coalition recognized *promotores*, or community health workers (CHWs), as essential change agents. However, all intervention components were oriented toward health promotion, and the coalition saw itself initially as the manager and representative of this program, not as an active advocate for community change.

#### Change in change agents

Policy impacts and environmental shifts were not fundamental to the structure of the coalition, despite its sponsorship by CDC and its awareness of the REACH 2010 change model. Though discussed in theory, policy was not originally the focus of the program. With considerable effort by the project director and UA partners, who were functioning formally as the project evaluators, the coalition slowly shifted its emphasis from program to policy. To do so, the coalition built on existing community strengths, such as the *promotoras*' ability, as client advocates embedded in the intervention communities, to expand their own role to include community advocacy (15). As with other coalitions, the *Promotora* Community Coalition was often asked, "What is the policy angle here?" by university partners during coalition meetings. The program/policy card game was first employed with this coalition.

#### Environmental shifts

To promote environmental shifts and longer-term policy changes in the three communities, the coalition worked with community leaders to design and implement public forums. The intention of the forums was to disseminate REACH 2010 results, solicit feedback, generate ideas, and obtain commitment from elected officials to support sustainable policy and program initiatives.

To date, each forum has tackled issues of health needs and priorities. These include the need for more physicians and pharmacies, improved transportation, and immunizations. Of significance was the participation of *promotores*, who advocated for changes in the environmental, occupational, and health behavior policies and programs in their communities. Using community input from the forums, the coalition's next steps are to review the forum results, recruit more community members to the coalition, identify short-term initiatives in each community, and develop a strategic plan (16).

#### Change in risk and protective factors

The three communities also witnessed environmental changes and shifts in local health behaviors. Participating community residents experienced positive outcomes from walking groups and cooking classes. These included improved physical activity, reduced fat intake, and increased consumption of fruits and vegetables. An improvement in the quality of diabetes care and provider–patient interactions was found in the health clinics (17). In addition, the members of the coalition acted as role models in the community, building health promotion and disease prevention awareness in the community (Figure).

### Yuma Special Action Group

#### Change in change agents

The Yuma, Arizona, Special Action Group (SAG) was formed in 1999 as a community coalition to address diabetes prevention and control through the Border Health Strategic Initiative (BHSI). BHSI was developed as a comprehensive and sustainable model for community-oriented chronic disease prevention and control (18). Based on the BHSI model and funding, the SAG's mission was to determine and prioritize community policy issues surrounding physical activity and healthy eating. The SAG efforts resulted in important changes to local school policies and the physical environment by changing community change agents. The coalition provided basic education to community members about risk factors for diabetes, identified community health issues, and developed and implemented action plans. The process of influencing policies encouraged community behavior change (19).

#### Environmental shifts

The first step toward development and implementation of the action plan was to form two subcommittees of change agents to develop plans based on the SAG's mission. One subcommittee developed an action plan to increase physical activity through advocating for more parks and walking paths. The second subcommittee addressed promoting healthier food choices in grocery stores and in schools. After meeting regularly, the two action plans were in place by June 2001.

As part of the physical activity action plan, the SAG organized three community forums. At the first forum, four grassroots neighborhood groups presented information about their ideas, challenges, and progress in park development and renovation. City and county officials offered information about funding and the need to work together on projects. A year later, a second forum for neighborhood groups and city and county officials gave team presentations about park construction, modeling the importance of working together on these projects. As a result of the first two forums and attendance at city and county public hearings, two neighborhood groups received Community Development Block Grants for county parks, and two new parks were developed in the small cities of Gadsden and Somerton. A third forum, held in 2004, emphasized increased awareness of and interest in park-building among the public and elected officials and the need to use the parks and recreational areas to improve healthy behaviors.

As part of the nutrition action plan, the SAG addressed product availability by southern Yuma County grocery stores. The SAG offered to provide healthy food promotion booths in stores, featuring food choices highlighted in community nutrition classes. This intervention was both programmatic and policy-driven. The programmatic objective was to change community behavior and customs, and the policy objective was to change the way the stores conducted business.

The health promotion booths were staffed by CHWs and other members of the SAG. Demonstrations were held at an average of three stores each month. The objective was to have more customers request healthier food choices and to have the stores stock and promote nutritious and low-fat foods such as low-fat and skim milk, diet soft drinks, yogurt, and fresh fruits and vegetables. After 8 months of negotiating with the major local store owner and presenting a written plan of the project, the store owner allowed the SAG to provide food samples and nutrition information in stores on the condition that the SAG purchase the food supplies in the stores and not dictate to customers what foods to buy or not to buy (19).

Outcomes of these efforts were hard to determine because the one owner of multiple stores, following customary industry-wide policy, refused to provide any quantitative sales data. These data were considered to be proprietary information. However, on the basis of interviews with store managers, we concluded that the sales of demonstration foods did increase in the week following the demonstration and continued for an undetermined time. Because of frequent changes in store stocking methods, we could not document actual changes in product placement on store shelves. Lack of project resources also prevented any regular, longer-term follow-up with store managers.

### Nogales/Santa Cruz County SAG

#### Change in change agents

The Nogales, Arizona, SAG was established in 1999 as a community coalition to address diabetes prevention and control through the BHSI (18). Engaging in the same process of self-education as the Yuma coalition, the Nogales SAG emphasized influencing planning for the county's physical environment (6). UA partners also assisted the Nogales SAG in working toward a policy focus.

The coalition encouraged change in community change agents by influencing the state's long-range development initiative through participation on the county's planning committee. Members learned about the Growing Smarter, Growing Plus program, a plan for growth in Nogales. They invited multiple speakers to SAG meetings, including the mayor, and advocated for needed recreational and park areas in the city. SAG members also encouraged the local newspaper to write articles on walking trails. Involvement by SAG members in city planning motivated developers, schools, and the city to consider the construction of bigger, better and improved parks, open spaces, and walking paths. By 2002, SAG members recognized the transition from programmatic to policy issues and began to see changes in local parks, such as the building of a skateboard park for youths.

#### Environmental shifts

Nogales SAG members advocated for a 0.75% sales tax increase to build a new hospital in Nogales and to provide funds for police and parks ("Changes in risk factors and protective factors," Figure). The process began with a meeting among city council members, local hospital administrators, and SAG members. The SAG encouraged the city council's involvement in the building of the new hospital. Nogales SAG members also went door to door, put up signs, and made presentations to local organizations to advocate for the initiative. In many cases, members educated the local public about the initiative, making sure that people voted in this first ever mail-in ballot during the November 8, 2005, election. The initiative passed with 55% of the votes. Without the SAG's and the hospital's help and dedication, the initiative very likely would have failed. Subgroups of the SAG are now working in three areas: monitoring the use of the new sales tax increase, promoting community walkability, and monitoring school health policy.

### Douglas SAG

#### Change in change agents

In 1997, Douglas, Arizona, residents joined forces with the UA partners and conducted a diabetes prevalence study to evaluate the health status and needs of the community. A programmatic coalition, the Diabetes Working Group, was established to bring resources to the community and raise awareness of the impact of diabetes in the community. In 2003, with funding from the UA Prevention Research Center, the group became the Douglas SAG and broadened its mission to include policy (6,20). Before becoming the Douglas SAG, members had conducted policy-related activities, such as meeting with city council members to discuss the health problems in the community. These activities, however, were not formalized in an advocacy action plan. The shift from program to policy was driven by the success of the BHSI in Yuma and Nogales and by the CAB's desire to see the model replicated in other border towns. UA partners promoted a policy agenda in the Douglas SAG with the idea of creating systems change that would improve chronic disease prevention and create change agents.

#### Environmental shifts

The transition from program to policy was timely, because coalition members were looking to combat diabetes using more systemic approaches, and they recognized the importance of policy activities. The Douglas SAG's shift emphasized changing physical activity and nutrition policy in local schools, where studies showed increasing rates of obesity. A nutrition policy drafted by the Douglas SAG was quickly implemented by the Douglas school district in all public schools and became a model for other communities in Arizona. While the Douglas SAG was drafting the nutrition policy, the U.S. Department of Agriculture mandated all public schools to develop nutrition standards as well as education and physical activity goals to promote student wellness, with a deadline of June 30, 2006. Because of the SAG's previous planning, the nutrition policy was approved and implemented a full year earlier than required. The Douglas SAG has continued to raise community awareness of communitywide health problems by regularly presenting to parent–student–teacher organizations and monitoring the local school district's nutrition policy.

### Southside Tucson

#### Change in change agents

The Sunnyside and Elvira Advocates for Health (SEAH) was established in 2004 as a grassroots neighborhood coalition that brought together the Sunnyside, Arizona, and Elvira, Arizona, neighborhood associations. Funding for this initiative came from the National Institutes of Health's (NIH's) Excellence in Partnerships for Community Outreach, Research in Health Disparities and Training (EXPORT) grant. The new coalition began by building strong partnerships with community residents and the university to determine areas of interest for collaboration on diabetes. During the course of discussion, the university worked with SEAH members to identify policy-related activities to target health problems in the neighborhoods. SEAH grew to include multiple partners, including service providers, members of the local school board, teachers, government officials and staff, business leaders, and UA partners (21). Without losing momentum or members, SEAH transitioned from a program-based to a policy-based coalition by embracing the need to change school and community-based policies to improve the community's health. As a result, three subcommittees were formed during 2005–2006 to address policy issues in the schools, local government, and local businesses.

#### Environmental shifts

In the schools, SEAH's specific advocacy resulted in rapid changes in the foods and beverages sold in vending machines (e.g., substitution of sugar-free soda and bottled water for high-sugar drinks). At the local government level, the planning and zoning division collaborated with SEAH to establish well-marked bike trails in the Sunnyside and Elvira neighborhoods. Among local businesses, SEAH worked with area supermarkets to provide healthy food demonstrations, which included a milk challenge that encouraged participants to buy and consume lower-fat milks. The leading grocery store in the area changed its milk promotion policy to apply to all types of milk rather than to whole milk only, as it had done previously.

## Interpretation

By applying the REACH 2010 Model of Change, we recognized that the community coalitions under study contributed to health behavior changes in their community not only by engaging in specific health promotion interventions, or targeted actions, but also by committing to a strategy that included policy advocacy. This commitment was the engine that drove the coalitions to focus on changing community change agents, resulting in community-based policy changes (6). Changes were made by first raising public health awareness among other health practitioners, community members, and elected officials. Then, coalition members worked with local government and schools to alter or create regulations and policies that promoted healthy behaviors. Members also advocated for increased funding for parks and fitness activities, changes in school curricula, removal or restocking of school vending machines, and sponsorship of regularly scheduled town hall meetings on health issues.

Moving from program to policy proved challenging in similar ways for all the coalitions. The impetus for this change first came from the UA's Prevention Research Center CAB general charge, which was promoted by the coalition's university partners and a few of the coalition's community members. It did not evolve as the result of a more organic process of consciousness change. Some coalition members had never worked on policy activities, and they were anxious about getting involved or advocating with local politicians and government. In fact, a tendency was observed among the five coalitions to revert to programmatic work and avoid policy advocacy, which required learning new skills, generating greater visibility, and introducing the potential for conflict and risk-taking. In addition, policy work tends to have a less immediate payoff than programmatic work. And, to sustain the momentum among coalition members for working on policy change, someone had to ask, "Where is the policy angle here?" Nevertheless, annual community coalition evaluations demonstrated that, although gaining policy and advocacy skills took time, the process built self-confidence and empowered coalition members to tackle broader community issues (12).

Of the lessons learned from the coalitions' movement from program management to policy action, the following are the most salient:

- Moving from program to policy may represent a paradigm shift in the health promotion culture of communities.
- The shift from health promotion to policy advocacy required skills that many coalition members lacked.
- The REACH 2010 Model of Change proved very useful in identifying and documenting the role played by changes in change agents.
- Timing and persistence are important. Coalitions advocating for environmental shifts and policy change recognized that each policy action has its own time or window of opportunity (14). The process of transition from program to policy varied by coalition, with each deciding its own agenda and tactics. In some cases, coalition members recognized that working on a particular policy issue was timely and salient to discussions and interests in the community, the local news media, and potential funders. In other coalitions, persistence and patience among members contributed to a willingness to wait until a window of opportunity was available in the community.
- The coalitions attempted to maximize inclusiveness and openness in their membership. They encouraged the recruitment of different actors outside traditional public health fields to participate in prioritizing policy targets, forming committees, and creating and implementing action plans.
- The practice of incorporating the university as a full partner and participant, beyond the role of the external technical expert or evaluator, contributed positively to community-based policy action. As a full partner, the UA was able to promote a policy focus for communities rather than to merely observe and measure. By the same token, it might be difficult to sustain the paradigm shift from program to policy without UA partners, and this may explain, in part, the coalitions' tendency to revert to a programmatic focus.
- Coalition members recognized that local involvement in and awareness of policy activities was important for sustaining long-term projects. Embedding programs and policies in the community by altering school regulations, city plans, and agency budgets supports sustainability.

Although the five coalitions demonstrated effectiveness in developing their members' knowledge and skills in advocacy and leadership, it remains to be seen whether and how these changes among change agents are transferable to the larger community and its policy makers, thereby contributing to long-term changes in health risk and protective factors (22). It is difficult to measure in terms of community health outcomes how the transition from program to policy has affected the five border communities. Significant, measurable change in health status may take many years. And at that point, we will be dealing with different actors, dynamics, and interests. Sustaining their momentum will be a continuing challenge to any and all community coalitions.

## Figures and Tables

**Table.** Coalition Successes and Challenges When Working on Policy and Environmental Shift Activities in United States–Mexico Border Communities

Coalition	**REACH 2010 Model of Change**	**Successes**	**Challenges**
The REACH 2010 *Promotora* Community Coalition	Community awareness and capacity Change in change agents Environmental shifts Changes in risk factors	Canvassed community to understand health needs and areas to target CHWs worked with community leaders to host community forums to disseminate results and advocate for improvements in community education Witnessed local health behaviors such as reduced fat intake and improved physical activity	Program was not implemented to focus on policy Continuous turnover in leadership interrupted progress toward goals and objectives
Yuma SAG	Change in change agents Environmental shifts	Developed action plans in physical activity and nutrition Hosted community forums on parks, recreation, and physical activity that provided a venue in which community organizations presented information to shape future policy Secured community development block grants for two parks Encouraged healthy behaviors through health food promotions	Programmatic focus tended to trump policy goals
Nogales/Santa Cruz County SAG	Change in change agents Environmental shifts	Participated in development planning for Santa Cruz County Provided leadership and door-to-door advocacy for passing a 0.75% sales tax increase to build a new hospital in Nogales and to increase funding for police and parks, including new walking and bike paths	Change in funding destabilized membership and leadership and reduced momentum
Douglas SAG	Change in change agents Environmental shifts	Emphasized changing physical activity and nutrition policies in local schools Raised awareness of community health problems by presenting information to parents and local schools Drafted a new nutrition policy, adopted by the school board, for the Douglas Unified School System Became the watchdog for implementation of the nutrition policy	Over time, the SAG narrowed its focus to working with schools, instead of involving the broader community
Southside Tucson: Sunnyside and Elvira Advocates for Health (SEAH)	Change in change agents Environmental shifts	Built strong partnerships with community residents and UA to identify policy issues Worked with local schools, government agencies, and neighborhoods to develop and implement a strategic plan to change foods sold in vending machines Worked with local grocery stores to change advertising for low-fat and skim milk	As the coalition grew and as subcommittees expanded activities, it was challenging to keep all members informed about the number of activities and policy initiatives

REACH indicates Racial and Ethnic Approaches to Community Health; CHWs, community health workers; SAG, Special Action Group; UA, University of Arizona.
